# Multizonal observational study conducted by clinical practitioners on evolocumab use in subjects with hyperlipidemia in Saudi Arabia and Kuwait: Results from the ZERBINI study

**DOI:** 10.1371/journal.pone.0278821

**Published:** 2023-01-20

**Authors:** Khalid Al Faraidy, Mousa Akbar, Mohamed Shehri, Mohammad Aljarallah, Gamal Abdin Hussein, Raja Dashti, Ahmad Al Qudaimi, Fahad Al Nouri, Zuhier Awan, Ahmed Essam, Alaa Emara

**Affiliations:** 1 KFMMC Cardiac Center, Interventional Cardiologist, King Fahd Military Medical Complex, Dharan, Saudi Arabia; 2 Cardiology Unit, Sabah Hospital, Kuwait City, Kuwait; 3 Cardiac Center Armed Forces Hospital Southern Region, Khamis Mushait, Saudi Arabia; 4 Sabah Al Ahmed Cardiac Center, Al-Amiri Hospital, Kuwait City, Kuwait; 5 Adult Cardiology Department, Cardiac Center North West Armed Forces King Salman Hospital, Tabuk, Saudi Arabia; 6 Sabah Al-Ahmad Cardiac Center, Amiri Hospital, Kuwait City, Kuwait; 7 Saud Al-babtain Cardiac Center, Dammam, Saudi Arabia; 8 Cardiovascular Prevention Unit, Prince Sultan Cardiac Centre, Riyadh, Saudi Arabia; 9 King Abdulaziz University, Clinical Biochemistry Jeddah University, Jeddah, Saudi Arabia; 10 Medical Affair Department, Amgen Middle East, Dubai, United Arab of Emirates; 11 Medical Department, Amgen Saudi, Saudi Arabia; Internal Medicine 1, ITALY

## Abstract

**Objectives:**

Dyslipidemia is a prevalent condition with significant morbidity and mortality across the world, including in the Arabian Gulf. The present study aimed to describe the characteristics of patients receiving evolocumab in clinical practice.

**Methods:**

ZERBINI was a multi-country, observational, retrospective/prospective study of subjects receiving evolocumab as part of routine clinical management of their hyperlipidemia. This regional publication reports on adult participants from Saudi Arabia and Kuwait who have had ≥1 dose of evolocumab before enrollment and ≤6 months’ prior exposure to evolocumab. Patient characteristics and treatment persistence data were collected in addition to baseline and follow-up data up to 12 months post-evolocumab initiation.

**Results:**

Overall, 225 patients were included from two sites, Saudi Arabia (N = 155) and Kuwait (N = 70). Mean age was comparable across sites and most patients had baseline coronary artery disease and/or hypertension. Baseline LDL-C levels (mean ± SD 3.6 ± 1.4 mmol/L in Saudi Arabia, 3.1 ± 1.4 mmol/L in Kuwait) were reduced by approximately 57%–62% in the first 6 months after evolocumab initiation (1.5 ± 1.2 mmol/L in Saudi Arabia [n = 63], 1.2 ± 0.8 mmol/L in Kuwait [n = 28]). This decrease was maintained over the 12-month follow-up period. Most patients achieved ACC 2018 LDL-C goals (<1.8 mmol/L; 74.6% in Saudi Arabia, 93.1% in Kuwait) and ESC 2019 LDL-C goals (<1.4 mmol/L; 66.7% in Saudi Arabia, 75.9% in Kuwait) in the first 6 months after evolocumab initiation. Medication persistence with evolocumab was high (up to 90.7%). Evolocumab had a favorable safety profile and no treatment-emergent adverse events were observed at either site.

**Conclusion:**

Evolocumab is an effective lipid-lowering treatment in local populations. LDL-C goal achievement is increased when evolocumab is added to background lipid-lowering therapy with high tolerability and persistence. Long-term follow-up and large-scale data are needed to further support these observations.

## Introduction

Cardiovascular diseases (CVD) are the leading noncommunicable cause of morbidity and mortality in the world, including in the Arabian Gulf region. The incidence of CVD continues to rise steadily and it is estimated that one-third of global deaths (nearly 18 million deaths) can be attributed to CVD [1,2]. The Arabian Gulf has amongst the highest rates of cardiovascular risk factors (e.g. diabetes mellitus, hypertension, dyslipidemia, metabolic syndrome, and smoking) in the world [2–5]. Elevated cholesterol, a leading modifiable risk factor for CVD, is considered a major threat to public health, particularly in highly affected regions [2]. The World Health Organization estimated the global prevalence of dyslipidemia to be 39% in 2008 [6]. The rising trend in dyslipidemia cases and related morbidity continues to be a concern around the world [2], and particularly in the Middle East [7]. In fact, over 4 million deaths were attributed to high low-density lipoprotein-cholesterol (LDL-C) levels globally in 2019, with the Middle East cited as having one of the highest burdens of elevated LDL-C [2]. The Arabian Gulf region also has a high prevalence of familial hypercholesterolemia (FH) compared with other regions, and a markedly low proportion of patients achieving LDL-C goals [8]. Previous studies in the region showed that LDL-C goals are not achieved in many patients, particularly among those who are at high risk of atherosclerotic cardiovascular disease (ASCVD) [9,10].

Mounting clinical trial, experimental, and genetic data indicate that elevated cholesterol levels can cause ASCVD [11–14]. Therefore, pharmacological approaches to reduce levels of total cholesterol, non-high-density lipoprotein-cholesterol (non-HDL-C) and, most importantly, LDL-C as a way to decrease the risk of CVD events are of increasing interest and importance [11–14]. The use of lipid-lowering interventions is also supported in low-risk early disease stages [15]. The introduction of proprotein convertase subtilisin/kexin type 9 (PCSK9) inhibitors (i.e. evolocumab and alirocumab) allowed the routine achievement of very low LDL-C levels in eligible patients and those who otherwise could not achieve lipid goals [16]. PCSK9 inhibitors ensure stable lowering of LDL-C levels and improvement of cardiovascular outcomes and are potentially superior to other lipid-lowering agents, without the risk of statin-related side effects [17,18].

The present study (ZERBINI) was designed to address the need for local real-world data on patients receiving evolocumab, and explored patient characteristics, the effectiveness of evolocumab in lowering LDL-C levels, and its safety. The study was conducted only in countries in the post-drug-approval setting and where local regulations allowed the study of a single treatment in clinical practice. Here, we present real-world data on the use of evolocumab for the management of hyperlipidemia from two Middle Eastern study sites: Saudi Arabia and Kuwait.

## Patients and methods

### Study design and patient population

ZERBINI was a multi-country, observational study of subjects receiving evolocumab as part of routine clinical management of their hyperlipidemia. The study, which had both retrospective and prospective aspects, collected real-world data on evolocumab use from Mexico, Brazil, Canada, Colombia, Saudi Arabia, and Kuwait. This publication focuses on the results of the ZERBINI study from Saudi Arabia and Kuwait; in these two sites, the first patient was enrolled in December 2018 and the last patient’s follow-up was completed in March 2021. Both male and female adults (≥18 years of age) were eligible to participate in this study if they were initiated on evolocumab at their physician’s discretion after August 1, 2017. Subjects also had to have had at least one dose of evolocumab before enrollment and ≤6 months’ prior exposure to evolocumab. This ensured that the study captured data as close as possible to a patient’s first dose of evolocumab, as well as enabling subjects from countries that gained early drug approval to be retrospectively included should they have less than 6 months’ exposure to evolocumab. Subjects with documented use of any PCSK9 inhibitors within 6 months prior to the initiation of evolocumab were excluded from the study.

### Data collection

**Fig 1:**
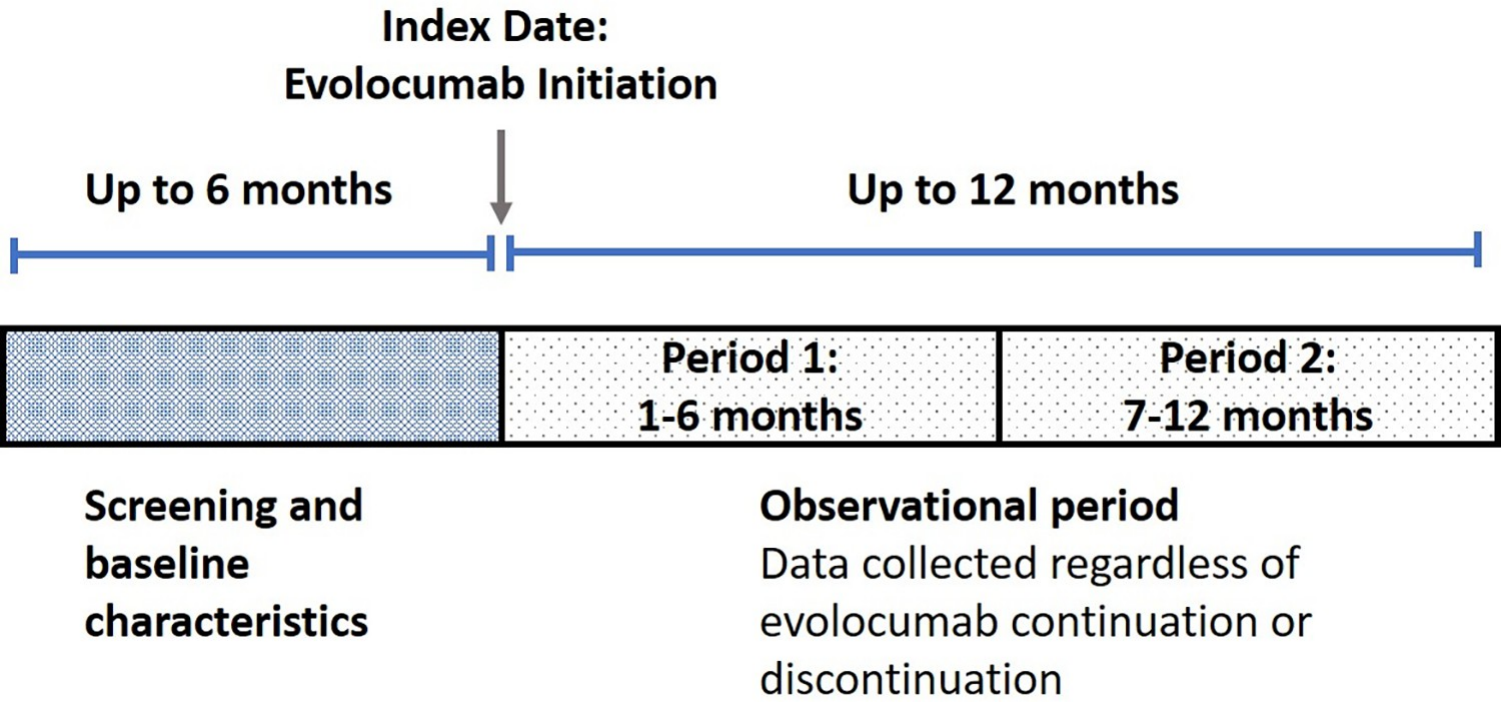
Flow diagram of study design.

Baseline data were collected at routine visits to the clinic during the 6 months prior to the index date (first dose of evolocumab) and follow-up data were collected at routine visits up to 12 months after the index date, regardless of evolocumab continuation or discontinuation (**Fig 1**). Variables captured for the baseline period included demographics (age, sex, and race); history of statin intolerance; FH status; diabetic status; use of lipid-modifying therapy; blood pressure; LDL-C, cholesterol, and hemoglobin A1C (HbA1C) levels; and the most recent LDL-C value. Additionally, a full cardiovascular history (conditions and events) was obtained for each subject.

LDL-C level, use of evolocumab (dose, frequency, switching to other PCSK9 inhibitors), use of lipid-modifying therapy after evolocumab initiation (type, dose, frequency, switching, augmentation), incidence of hospitalization (reason for admission/final diagnosis, admission date, and discharge date), and incidence of physician visits were also tracked over the 12 months of follow-up after evolocumab initiation. To reduce unnecessary burden on sites and to improve reporting of relevant safety data, only those adverse events considered by the investigator to be causally related to evolocumab administration (treatment-emergent adverse events) were included. Similarly, only data relating to hospitalizations that resulted from an adverse drug reaction were collected.

### Statistical analysis

The statistical analysis was descriptive in nature and stratified by country, with no statistical comparisons and no imputations for missing data. Patient demographic and clinical characteristics were summarized using the number of subjects, mean, median, standard deviation (SD), 25th percentile, 75th percentile, minimum and maximum values for numeric variables, and frequency distributions (frequency and percentage) for categorical variables. In addition, for history of CVD and FH status, 95% confidence intervals were computed using the Wald method, or the Clopper–Pearson method when the observed counts fell below 5. When considering baseline characteristics, for each subject, the mean baseline LDL-C value, other cholesterol values, and HbA1c value were considered to be the last measurement taken prior to the initiation of evolocumab therapy. Changes in LDL-C post-initiation of evolocumab were summarized over two time periods within the 12 months of follow-up. Period 1 included data from patients who had at least one follow-up record between Months 1 and 6, and period 2 included data from patients who had at least one follow-up record between Months 7 and 12 (**Fig 1**). For patients with multiple visits during each of the two follow-up periods, the mean LDL-C values were computed and used to represent that patient. Absolute and percent changes from baseline were computed and summarized in the same way as numeric variables. In addition, the number and percentage of patients with post-baseline LDL-C level <50 mg/dL (<1.3 mmol/L), <55 mg/dL (<1.4 mmol/L), and <70 mg/dL (<1.8 mmol/L) were computed for each of the two time periods. Persistence reflects the proportion of patients who received evolocumab for the entire follow-up period after initiation, and who had no dose gaps exceeding the allowable based on the evolocumab dosing instructions (56 consecutive days). Based on this, patients were categorized as either persistent or nonpersistent [19,20]. Patients were excluded from persistence calculations if they did not complete the study for reasons that were not evolocumab related (N = 32): patient request (n = 9), lost to follow-up (n = 8), discontinuation unrelated to evolocumab (n = 15). Patients who withdrew from the study due to an adverse event, death, or unknown reasons were captured as nonpersistent.

Lastly, treatment exposure and safety were computed and summarized in a similar manner as that used for the demographic and clinical characteristics. Data were analyzed using IBM SPSS version 27.0 (IBM Corp. Released 2020. IBM SPSS Statistics for Windows, Armonk, NY: IBM Corp).

### Ethics considerations

This study was approved by the relevant ethics committees of all participating centers. All participants completed a written informed consent form before enrollment. Data were anonymized prior to analysis.

## Results

### Baseline patient characteristics

Overall, 225 patients were included in the study: 155 patients from Saudi Arabia and 70 patients from Kuwait. Patients’ mean age (55.4 ± 11.1 years in Saudi Arabia, 56.7 ± 9.6 years in Kuwait) and race distributions were comparable between the two countries; 78.1% of patients in Saudi Arabia and 51.4% of patients in Kuwait were male (**Table 1**).

**Table 1:** Patient demographics in Saudi Arabia and Kuwait.

		Saudi Arabia (N = 155)	Kuwait (N = 70)
**Age, years**			
	*Mean ± SD*	55.4 ± 11.1	56.7 ± 9.6
** **	*Median (Q1*–*Q3)*	56 (47–63)	58 (49–64)
** **	*Range*	24–84	32–73
**Gender**			
	*Male*, *n (%)* *Female*, *n (%)*	121 (78.1) 34 (21.9)	36 (51.4) 34 (48.6)
**Race**			
** **	*White*, *n (%)*	77 (49.7)	31 (44.3)
** **	*Other*, *n (%)*	78 (50.3)	39 (55.7)

Q: Quartile; SD: Standard deviation.

With regards to baseline clinical characteristics (**Table 2**), the vast majority of patients in both countries had a concomitant cardiovascular condition. The most commonly reported baseline cardiovascular conditions in Saudi Arabia were coronary artery disease (CAD) (87.7%), hypertension (76.1%), and myocardial infarction (61.9%). In Kuwait, hypertension was the most prevalent (72.9%), followed by CAD (65.7%) and myocardial infarction (51.4%). The prevalence of diabetes was similar in Saudi Arabia (62.5%) and Kuwait (58.5%). Overall, 16.1% and 5.7% of patients were diagnosed with FH in Saudi Arabia and Kuwait, respectively. The vast majority of those diagnosed with FH were heterozygous. Baseline LDL-C levels were 3.6 ± 1.4 mmol/L in Saudi Arabia and 3.1 ± 1.4 mmol/L in Kuwait. Overall, 94.8% and 85.7% of patients were receiving statins at baseline in Saudi Arabia and Kuwait, respectively. Approximately 14% of patients from both sites were on a low-to-moderate dose of statin; 81.3% and 70.0% of patients from Saudi Arabia and Kuwait, respectively, were receiving high-dose statins. History of statin intolerance was higher in Kuwait (37.1%) than in Saudi Arabia (7.7%). Ezetimibe use was higher in Saudi Arabia (31.0%) than in Kuwait (21.4%). At treatment initiation, all patients from both sites were receiving 140 mg of evolocumab through a pre-filled pen (SURECLICK^®^ injector) every 2 weeks.

**Table 2:** Baseline characteristics of patients in Saudi Arabia and Kuwait.

		Saudi Arabia (N = 155)	Kuwait (N = 70)
**CV history**			
	*Subjects with any CV condition*, *n (%)*	148 (95.5)	61 (87.1)
	*95% CI*	91.0, 97.8	77.3, 93.1
**CV condition, n (%)**			
	*Coronary artery disease*	136 (87.7)	46 (65.7)
	*Hypertension*	118 (76.1)	51 (72.9)
	*Myocardial infarction*	96 (61.9)	36 (51.4)
	*Congestive heart failure*	16 (10.3)	3 (4.3)
	*Atrial fibrillation*	7 (4.5)	4 (5.7)
	*Transient ischemic attack*	6 (3.9)	2 (2.9)
	*Peripheral artery disease*	5 (3.2)	2 (2.9)
	*Cerebrovascular accident*	4 (2.6)	8 (11.4)
	*Deep vein thrombosis*	2 (1.3)	0 (0.0)
	*Intermittent claudication*	1 (0.6)	3 (4.3)
**Diabetes, n (%)**			
	*Diabetic*	97 (62.5)	41 (58.5)
	*Type 1*	3 (1.9)	1 (1.4)
	*Type 2*	93 (60.0)	40 (57.1)
	*Non-diabetic*	58 (37.4)	29 (41.4)
	*Unknown*	1 (0.6)	
**FH, n (%)**			
	*Diagnosed*	25 (16.1)	4 (5.7)
	*95% CI*	11.2, 22.7	2.2, 13.8
	*Not diagnosed*	32 (20.6)	13 (18.6)
	*Unknown*	98 (63.2)	53 (75.7)
**Among those diagnosed with FH, n (%)**			
	*Heterozygous*	19 (12.3)	4 (5.7)
	*Homozygous*	6 (3.9)	0 (0.0)
**Method of FH diagnosis, n (%)**			
	*Simon Broome*	3 (1.9)	0 (0.0)
	*Dutch Lipid Clinic Network*	19 (12.3)	0 (0.0)
	*Genetic testing*	1 (0.6)	0 (0.0)
	*LDL-C*	5 (3.2)	0 (0.0)
	*Other*	1 (0.6)	4 (5.7)
**LDL-C, mmol/L**			
	*n*	149	62
	*Mean ± SD*	3.6 ± 1.4	3.1 ± 1.4
	*Median (Q1–Q3)*	3.2 (2.8–4.1)	2.8 (2.3–3.6)
	*Range*	0.7–9.5	1.0–9.9
**HbA1c with diabetes, mmol/mol**			
	*n*	74	26
	*Mean ± SD*	8.6 ± 1.8	8.3 ± 2.1
	*Median (Q1–Q3)*	8.5 (7.1–9.4)	7.6 (7.0–9.8)
	*Range*	6.0–13.8	5.4–12.8
**HbA1c without diabetes, mmol/mol**			
	*n*	30	16
	*Mean ± SD*	6.0 ± 0.8	5.7 ± 0.5
	*Median (Q1–Q3)*	5.9 (5.6–6.3)	5.7 (5.1–6.1)
	*Range*	4.8–9.1	4.9–6.5
**History of statin intolerance, n (%)**		12 (7.7)	26 (37.1)
**Baseline blood pressure, mm/Hg**			
	*Diastolic*	77.7	76.6
	*Systolic*	133.8	137.3
**Statin use, n (%)**			
	*Overall*	147 (94.8)	60 (85.7)
	*Low-moderate intensity* ^*a*^	21 (13.5)	10 (14.3)
	*High intensity* ^*b*^	126 (81.3)	49 (70.0)
**Ezetimibe use, n (%)**		48 (31.0)	15 (21.4)
**Evolocumab use at initiation, n (%)**			
	*140 mg Q2W*	155 (100.0)	70 (100.0)
	*Pre-filled syringe*	0 (0.0)	0 (0.0)
	*Autoinjector*	0 (0.0)	0 (0.0)
	*SURECLICK*^*®*^ *injector 1*.*5*	155 (100.0)	70 (100.0)
	*420 mg QM*	0 (0.0)	0 (0.0)

^a^Atorvastatin ≤20 mg, pitavastatin ≤20 mg, pravastatin 20 mg, pravastatin 40 mg; rosuvastatin 20 mg, simvastatin 40 mg

^b^Atorvastatin 40 mg, atorvastatin 80 mg, rosuvastatin 40 mg, rosuvastatin 20 mg.

CI: Confidence interval; CV: Cardiovascular; FH: Familial hypercholesterolemia; HbA1c: Hemoglobin A1C; LDL-C: Low-density lipoprotein-cholesterol; Q: Quartile; Q2W: Every 2 weeks; QM: Every month; SD: Standard deviation.

### Effectiveness of evolocumab

Patients could have had a visit in the first period but not in the second period (and vice versa), and LDL-C results were not available for all study participants during the follow-up period. Evolocumab was effective in reducing LDL-C levels in both patient populations (**Table 3**). In Saudi Arabia, there was a 57.4% decrease from baseline in LDL-C levels in the first 6 months after evolocumab initiation (n = 63), and a 47.9% decrease in the 7 to 12 months after evolocumab initiation (n = 79). At this site, 74.6% of patients achieved the American College of Cardiology (ACC) 2018 LDL-C goals (<1.8 mmol/L) and 66.7% achieved the European Society of Cardiology (ESC) 2019 LDL-C goals (<1.4 mmol/L) in the first 6 months after evolocumab initiation. In Kuwait, there were comparable decreases in LDL-C levels (61.7% in the first 6 months after evolocumab initiation, and 57.9% in the 7 to 12 months after evolocumab initiation). Most patients (93.1%) from Kuwait achieved the ACC 2018 LDL-C goals (<1.8 mmol/L) and 75.9% achieved the ESC 2019 LDL-C goals (<1.4 mmol/L) in the first 6 months after evolocumab initiation. Overall, 63.5% and 72.4% of patients from Saudi Arabia and Kuwait, respectively, achieved LDL-C levels below 1.3 mmol/L in the first 6 months after evolocumab initiation. No complications were observed with the achievement of low LDL-C levels. Ezetimibe and statin use remained relatively stable during the 12-month follow-up period.

**Table 3:** Evolocumab effectiveness in the 12-month follow-up period after treatment initiation in Saudi Arabia and Kuwait.

			At baseline	Post-initiation of evolocumab (Month 1–6)	Post-initiation of evolocumab (Month 7–12)
**Saudi Arabia**	**Number of patients still on evolocumab for at least part of that period**		N = 155	N = 155	N = 150
	**Number of patients receiving statins, n (%)**				
		*Overall*	147 (94.8)	_	_
		*Low-to-moderate intensity* ^*a*^	21 (13.5)	_	_
		*High-intensity* ^*b*^	126 (81.3)	_	_
	**Number of patients with LDL-C results**		n = 149	n = 63	n = 79
		*Mean (SD) LDL-C level*, *mmol/L*	3.6 (1.4)	1.5 (1.2)	1.8 (1.3)
		*Mean (SD) absolute change from baseline*, *mmol/L*		-2.2 (1.4)	-1.9 (1.6)
		*Mean (SD) % change from baseline*, *mmol/L*		-57.5 (32.0)	-47.9 (39.1)
	Post-baseline LDL-C <50 mg/dL (<1.3 mmol/L)			63.5%	49.4%
	Post-baseline LDL-C <55 mg/dL (<1.4 mmol/L)			66.7%	50.6%
	Post-baseline LDL-C <70 mg/dL (<1.8 mmol/L)			74.6%	67.1%
**Kuwait**	Number of patients still on evolocumab for at least part of that period		N = 70	N = 68	N = 63
	Number of patients receiving statins, n (%)				
		*Overall*	60 (85.7)	_	_
		*Low-to-moderate intensity* ^*a*^	10 (14.3)	_	_
		*High-intensity* ^*b*^	49 (70.0)	_	_
	Number of patients with LDL-C results		n = 62	n = 29	n = 26
		*Mean (SD) LDL-C level*, *mmol/L*	3.1 (1.4)	1.2 (0.8)	1.6 (1.2)
		*Mean (SD) absolute change from baseline*, *mmol/L*		-2.0 (1.1)	-1.7 (0.7)
		*Mean (SD) % change from baseline*, *mmol/L*		-61.7 (23)	-57.9 (23.4)
	Post-baseline LDL-C <50 mg/dL (<1.3 mmol/L)			72.4%	53.8%
	Post-baseline LDL-C <55 mg/dL (<1.4 mmol/L)			75.9%	57.7%
	Post-baseline LDL-C <70 mg/dL (<1.8 mmol/L)			93.1%	69.2%

^a^Atorvastatin ≤20 mg, pitavastatin ≤20 mg, pravastatin 20 mg, pravastatin 40 mg; rosuvastatin 10 mg; rosuvastatin 20 mg, simvastatin 40 mg; ^b^Atorvastatin 40 mg, atorvastatin 80 mg, rosuvastatin 40 mg, rosuvastatin 20 mg.

LDL-C: Low-density lipoprotein-cholesterol; SD: Standard deviation.

### Treatment persistence and safety

Of the 225 patients participating in the study from both sites (Saudi Arabia and Kuwait), 193 were included in the persistence calculations: 175 (90.7%) of patients were persistent and 18 (9.3%) were found to be nonpersistent. Reasons for nonpersistence among these 18 patients were adverse drug reactions (n = 2), requirement for an alternative therapy (n = 1), death (n = 1), missed doses for >56 consecutive days (n = 1), and other/unknown (n = 13).

There were no treatment-emergent adverse drug reactions, serious adverse drug reactions, or adverse reactions leading to evolocumab discontinuation recorded in either country.

## Discussion

The present analysis of the ZERBINI study results from Saudi Arabia and Kuwait showed that evolocumab was highly effective in reducing LDL-C levels in a real-world setting. Evolocumab treatment reduced LDL-C levels by approximately 2 mmol/L during the 12-month follow-up period. As a result, a high proportion of patients achieved treatment goals despite previous use of high-intensity statins and ezetimibe. In the first 6 months after evolocumab initiation, the ACC 2018 LDL-C goal (<1.8 mmol/L) was achieved by 74.6% of patients in Saudi Arabia and 93.1% of patients in Kuwait, while the ESC 2019 LDL-C goal (<1.4 mmol/L) was achieved by 66.7% of patients in Saudi Arabia and 75.9% of those in Kuwait. Treatment persistence was high and comparable with the rate reported for another country participating in the ZERBINI study (i.e. Canada [20]). Evolocumab was also found to have a favorable safety profile in this study population, with no treatment-emergent adverse drug reactions recorded, even in patients achieving very low LDL-C levels (<1.3 mmol/L).

Although the lipid-lowering efficacy of PCSK9 inhibitors is supported by a wealth of evidence in dyslipidemia [16–18,21], the use of PCSK9 inhibitors remains low in the Arabian Gulf for patients with FH and those with highly elevated LDL-C levels. Similarly, the achievement of LDL-C goals remains challenging [8–10]. Starting from baseline LDL-C levels of 3.4 mmol/L in Saudi Arabia and 3.1 mmol/L in Kuwait, evolocumab-treated patients in this study achieved mean LDL-C levels of 1.2 and 1.5 mmol/L in the first 6 months after treatment initiation, and 1.6 and 1.8 mmol/L 7–12 months after treatment initiation, respectively. This translates to an average of 52.7%–60% decrease in LDL-C levels over the 12-month follow-up period. The effectiveness of evolocumab in both patient populations is consistent with the available evidence from other reports. For example, the ZERBINI study results from Canada showed a 59% reduction in LDL-C levels over the 12-month study period [20]. LDL-C reduction rates with evolocumab in clinical trials (FOURIER study [22] and the RUTHERFORD-2 FH patient cohort [23]), as well as real-world evidence (HEYMANS European study [24]), are also comparable. Furthermore, the open-label extension of the OSLER-1 trial showed that the use of evolocumab in addition to standard of care safely and persistently reduced LDL-C levels by approximately 56% over the 5-year follow-up period [25].

The present study supports the high predictability of evolocumab in achieving a significant reduction in LDL-C levels. The results also support the real-world role of evolocumab in accordance with international [26,27] and Middle Eastern [7] guideline recommendations for the use of PCSK9 inhibitors in very high-risk patients who are unable to achieve LDL-C goals with statins and ezetimibe. Baseline LDL-C levels were elevated in both study cohorts (3.4 mmol/L in Saudi Arabia and 3.1 mmol/L in Kuwait) despite high-intensity statin and ezetimibe use at baseline. Regardless, the majority of patients on evolocumab achieved both the ACC 2018 and ESC 2019 LDL-C treatment goals (<1.8 mmol/L and <1.4 mmol/L, respectively) in the first 6 months after treatment initiation. This is consistent with data from other studies reporting 67% to 87% of patients achieving LDL-C <1.8 mmol/L during evolocumab treatment [20,22,25]. Moreover, 49%–54% of patients in the present study achieved very low LDL-C levels (<1.3 mmol/L) without any reported imbalances in adverse events compared to those with higher LDL-C values. By helping patients achieve recommended LDL-C goals, the use of evolocumab could be protective against future cardiovascular events in very high-risk patients. A meta-analysis confirmed that evolocumab significantly decreased both LDL-C levels and the incidence of cardiovascular adverse events in patients at high cardiovascular risk [28]. This was consistent with the findings from the FOURIER trial, which demonstrated a significant reduction in the risk of cardiovascular events with evolocumab [22]. This is of particular interest to populations of Arabian Gulf countries, which have high prevalence of cardiovascular risk factors [2–5] and high proportions (approximately 70%–85%) of very high-risk patients not achieving LDL-C goals with lipid-lowering therapy [9,10,29]. The cost-effectiveness of evolocumab for patients with ASCVD or heterozygous FH has been demonstrated in Saudi Arabia [30], further supporting it as a good treatment option for lipid control in these local populations.

A slight difference in evolocumab efficacy between the first 6 months of treatment and 7–12 months following treatment initiation was noted in the ZERBINI study results from Canada [20]; a similar effect was also observed in the Saudi Arabian and Kuwait populations. We concur that this difference could be related to variable baseline patient characteristics (i.e. baseline LDL-C level was lower in some patients), variation in patient physiology, or small sample size. However, it is more likely due to changes in background lipid-lowering therapy than variance in evolocumab efficacy over time. Nonadherence to lipid-lowering therapy is widely reported and has a significant impact on treatment success [31–33]. Clinical evidence supports the consistency of evolocumab’s lipid-lowering effect, as comparable reductions in LDL-C levels with evolocumab use were observed across racial/ethnic groups in both short-term (12 weeks) and long-term (1–5 years) studies [34].

Overall, patients in both countries exhibited high persistence to evolocumab treatment, consistent with the existing body of evidence [20,25]. The favourable safety profile of evolocumab could be a major contributing factor to the observed drug persistence rates. No treatment-emergent adverse drug reactions were recorded in the present study. Other studies also report few adverse events related to evolocumab, and a decreasing trend in injection-site reactions with longer duration of evolocumab use [20,22,25]. In contrast, persistence and adherence rates to statins are relatively low, even after the occurrence of cardiovascular events [35,36], further highlighting the need to include PCSK9 inhibitors such as evolocumab in lipid management strategies.

In the present study, some patients were lost to follow-up in Kuwait and Saudi Arabia due to the effects of the COVID-19 pandemic and lockdown measures. In fact, the follow-up period of the ZERBINI study in Kuwait and Saudi Arabia largely coincided with the first year of the COVID-19 pandemic, when restrictions were at their tightest. A cross-sectional study from Saudi Arabia reported that more than half of patients missed follow-up visits for their chronic condition during the pandemic [37]. Clinical experience during the global pandemic and the ensuing restrictions in Arab countries, including in the Gulf, showed delayed outpatient follow-up, medication interruptions, and decreased access to non-emergency healthcare [38,39]. Changes in pharmacological adherence due to the COVID-19 pandemic have been documented in several studies worldwide, across a wide range of chronic diseases [40–42].

Another issue observed in clinical practice in Saudi Arabia and Kuwait is the suboptimal frequency of LDL-C testing. LDL-C levels were only tested for ≤50% of patients receiving evolocumab over the 12-month follow-up period in the present study. This could explain the low proportions of patients achieving LDL-C treatment goals in the Middle East region [9,10], as evidence correlates lipid testing with therapy initiation or escalation [43]. Therefore, frequent monitoring of lipid levels should be encouraged, in accordance with guideline recommendations for secondary prevention or in patients at very high cardiovascular risk [26]. This will ensure optimization of lipid management and provision of more potent drugs, such as PCSK9 inhibitors, to patients who are in need and eligible, but whose poor level of lipid control on lipid-lowering therapy would otherwise remain undetected.

## Conclusions

The ZERBINI study provides real-world evidence from Saudi Arabia and Kuwait on the effectiveness of evolocumab in reducing LDL-C levels over the 12 months after drug initiation. Baseline LDL-C levels were elevated despite the use of high-intensity statins and/or ezetimibe in the majority of the participants. Addition of evolocumab ensured highly pronounced reduction in LDL-C levels (52.7%–60% during the 12-month follow-up period), with more than two-thirds of patients achieving the LDL-C goals (<1.4 mmol/L or <1.8 mmol/L) in the first 6 months after evolocumab initiation. High medication persistence was also observed. Evolocumab was found to have a favorable safety profile, with no treatment-emergent adverse drug reactions recorded at either of the two study sites. Our results support the use of evolocumab for improving the management of lipid levels in these local patient populations, where goal achievement remains poor. Long-term follow-up data and larger-scale studies are necessary to establish statistical inferences and further inform clinical practice.
